# Approaches to kidney replacement therapies—opportunities and challenges

**DOI:** 10.3389/fcell.2022.953408

**Published:** 2022-07-22

**Authors:** Biao Huang, Zipeng Zeng, Chennan C. Zhang, Megan E. Schreiber, Zhongwei Li

**Affiliations:** ^1^ USC/UKRO Kidney Research Center, Division of Nephrology and Hypertension, Department of Medicine, Keck School of Medicine, University of Southern California, Los Angeles, CA, United States; ^2^ Deptartment of Stem Cell Biology and Regenerative Medicine, Keck School of Medicine, University of Southern California, Los Angeles, CA, United States

**Keywords:** pluripotent stem cells, bioartificial kidney, decellularizalion, kidney organoid, xenotransplant, interspecies chimera, genome editing, bioengineering (general)

## Abstract

One out of seven people develop chronic kidney disease (CKD). When kidney function continues to decline, CKD patients may develop end-stage renal disease (ESRD, or kidney failure). More than 2 out of 1,000 adults develop ESRD and these patients must live on dialysis or get a kidney transplant to survive. Each year, more than $51 billion is spent to treat patients with ESRD in the United States. In addition, ESRD greatly reduces longevity and quality of life for patients. Compared to dialysis, kidney transplant offers the best chance of survival, but few donor organs are available. Thus, there is an urgent need for innovative solutions that address the shortage of kidneys available for transplantation. Here we summarize the status of current approaches that are being developed to solve the shortage of donor kidneys. These include the bioartificial kidney approach which aims to make a portable dialysis device, the recellularization approach which utilizes native kidney scaffold to make an engineered kidney, the stem cell-based approach which aims to generate a kidney *de novo* by recapitulating normal kidney organogenesis, the xenotransplantation approach which has the goal to make immunocompatible pig kidneys for transplantation, and the interspecies chimera approach which has potential to generate a human kidney in a host animal. We also discuss the interconnections among the different approaches, and the remaining challenges of translating these approaches into novel therapies.

## Introduction

Kidneys filter our blood and remove wastes, acid and extra fluid from our bodies, and help maintain a healthy balance of water, salt, pH, and minerals in our blood. In addition, by producing hormones, kidneys regulate our blood pressure and red blood cell production and keep our bones strong. Humans and other animals cannot live without this essential organ.

During the embryonic and fetal stages, kidneys naturally form through the process of organogenesis. Overall, mouse and human kidney organogenesis share considerable similarities (McMahon, 2016; Lindström et al., 2018a; Lindström et al., 2018b; Lindström et al., 2018c). In the mouse at around embryonic day 10.5 (E10.5), part of the Wolffian duct (named “ureteric bud” or UB) invades the surrounding mesenchymal tissue (named “metanephric mesenchyme” or MM), marking the initiation of kidney organogenesis. A similar invasion of UB into MM is also observed in the human at around 5 weeks of gestational age. The developing kidney then enters the next phase with the expansion of UB and MM, coupled with repetitive mutual induction between MM and UB. Eventually, over a period of around 10 days in the mouse, and around 31 weeks in the human, the MM gives rise to all the nephrons, the functional units of the kidney (∼14,000 nephrons in a mouse kidney, and ∼1 million nephrons in a human kidney). Meanwhile, the UB undergoes extensive branching morphogenesis to generate a tree-like collecting duct (CD) network for processing and transporting the urine generated from the nephrons to the bladder.

**FIGURE 1:**
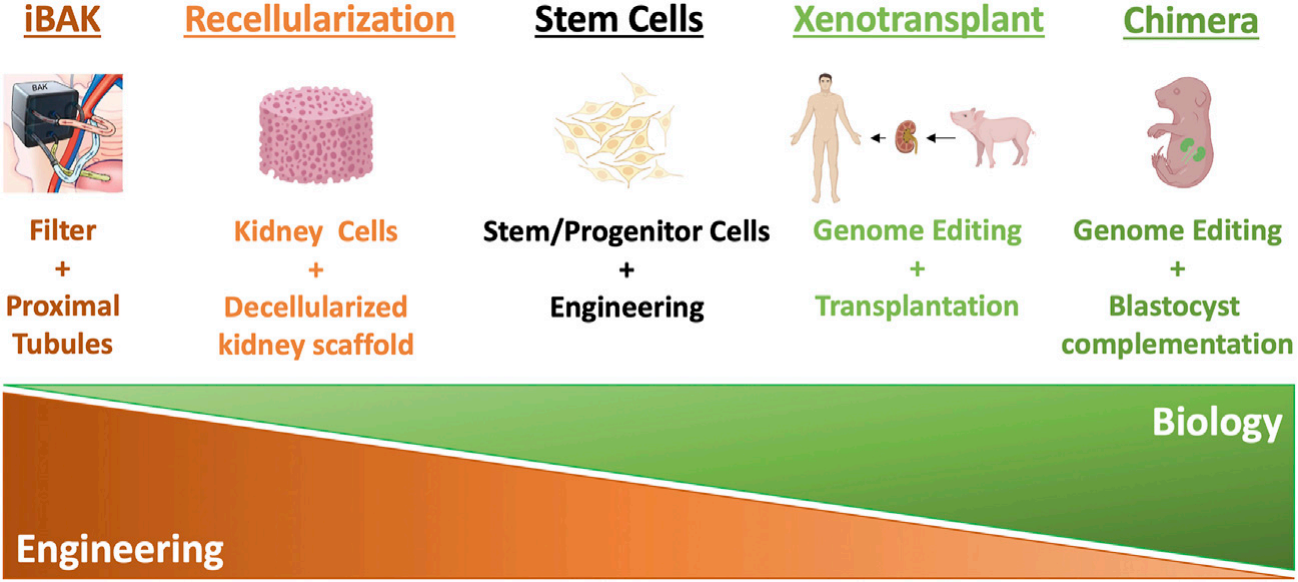
Summary of different kidney replacement approaches.

However, kidney function declines with age, upon injury, or under various disease conditions, and the adult kidney has a very limited ability to regenerate itself and recover. Therefore, diminishing kidney function tends to progress down a one-way street, and it is difficult to reverse course. When kidney function decreases to a certain point, a patient develops chronic kidney disease (CKD). According to Centers for Disease Control and Prevention (CDC), 15% of the adults in the United States, or 37 million Americans, have CKD (https://www.cdc.gov/kidneydisease/publications-resources/ckd-national-facts.html). In the clinical setting, doctors describe the progression of CKD from stages 1–5 in increasing order of severity (Chen et al., 2019). These stages are based on glomerular filtration rate (GFR), an indicator of kidney function that calculates how fast wastes are filtered and extra fluids removed from the blood, measured in milliliters per minute (ml/min). With impaired kidney function, GFR drops correspondingly. During stages 4–5 (GFR <30), patients experience signs and symptoms, including nausea, fatigue, drowsiness, weakness, headache, loss of appetite, itching, muscle cramps, hypertension, volume overload, anemia, bone, mineral, and electrolyte abnormalities, etc. Drug treatments and a low-protein, low-salt diet can help manage signs and symptoms and slow but often not stop the progression of CKD, for which there is no cure (Chen et al., 2019). When GFR drops to less than 15 ml/min, patients reach stage 5, or ESRD, in which remaining kidney function is not enough to support life. According to the 2021 Annual Data Report from United States Renal Data System (USRDS), currently, more than 809,103 Americans live with ESRD (more than 2 in every 1,000 people, https://adr.usrds.org/2021). ESRD patients need either dialysis or a kidney transplant to survive. On an annual basis, more than $51 billion is spent to treat patients with ESRD in the United States, accounting for more than 7% of the Medicare budget. In the most common form of dialysis, hemodialysis, a machine filters wastes from the blood, which is then returned to the body. Patients typically spend 3–5 h per dialysis session 3 days per week in the dialysis center, dramatically reducing their quality of life. Moreover, even with this treatment, only 35%–50% of hemodialysis patients survive for 5 years. Kidney transplant carries a more favorable 5-year survival rate of more than 80%, but there is a severe shortage of donor organs. In 2021, 90,483 Americans were on the waiting list for donor kidneys, but only 24,670 received a transplant (https://www.organdonor.gov). It is estimated that 13 Americans die each day while waiting for a kidney transplant. Thus, there is an urgent need for innovative solutions that address the shortage of kidneys available for transplantation. In this review, we introduce the concept and summarize the progress of the current approaches to solving the shortage of donor kidneys (summarized in Figure 1). We also discuss the advantages and challenges of the approaches (summarized in Table 1).

**TABLE 1:** Advantages and challenges of different kidney replacement approaches.

	IBAK	Recellularization	Stem cells	Xenotransplant	Chimera
Advantages	No immune rejection	Native kidney scaffold	No immune rejection	Functionality Maturity	Grow kidney *in vivo*
	No ethical concerns	No ethical concern	No ethical concerns		
Challenges	Cell source	Cell source	Scalability	Immunogenicity	Xenotransplant
	Long-term efficacy	Recellularization	Urine excretion	Long-term efficacy	Safety concerns
	Durability	Long-term efficacy	Vasculature	Safety concerns Ethical concerns	Ethical concerns

## Bioartificial kidney—making a portable dialysis device

Bioartificial kidneys are built upon dialysis devices. Like dialysis, bioartificial kidneys employ artificial hemofilters to filter wastes from the blood. Furthermore, a biological component consisting of kidney’s proximal tubule cells is added to enhance the performance of the dialysis device, aiming at recapitulating not only the filtration function of the kidney, but also most of the kidney’s reabsorption and excretion functions (Humes et al., 2014; Salani et al., 2018). This review focuses on this type of bioartificial kidney. It is of note that other wearable artificial kidney devices aiming at miniaturizing dialysis device, without a biological component, are also being actively developed and are reviewed elsewhere (Salani et al., 2018).

The development of bioartificial kidneys started with a pioneering study from Humes and colleagues with the generation of an extracorporeal renal tubule assist device (RAD) (Humes et al., 1999a). This RAD consisted of about 10^8^ renal proximal tubule cells grown as confluent monolayer along the inner surface of one standard hemofiltration cartridge. RAD was then connected to a conventional hemofilter to assemble the bioartificial kidney. Both *in vitro* and *in vivo* studies of the system showed that the renal cells retained their normal functions, such as metabolic activities, active transport properties, and key endocrine processes (Humes et al., 1999a; Humes et al., 1999b; Fissell et al., 2003). This RAD-based bioartificial kidney was then tested in FDA-approved clinical trials for the treatment of acute renal failure (ARF), for which the mortality rate exceeding 70%, despite hemodialysis or continuous renal replacement therapy (CRRT). In an initial Phase I/II clinical trial, safety of RAD device was demonstrated for up to 24 h, with maintained viability, durability, and functionality (Humes et al., 2004). In a follow-up multicenter, randomized, Phase II clinical trial involving 58 patients who had ARF and required CRRT, patients with CRRT + RAD treatment had dramatically reduced mortality rate (33% CRRT + RAD vs. 61% CRRT-only at day 28). The risk for death in the CRRT + RAD group was estimated to be 50% of the CRRT-only group (Tumlin et al., 2008). These results highlight the potential of RAD device in the treatment of ARF.

Based on the RAD system, Roy, Fissell, and colleagues from University of California, San Francisco (UCSF) and Vanderbilt University aim to further miniaturize the device size in order to create an implantable bioartificial kidney (iBAK) as a permanent solution to ESRD (https://pharm.ucsf.edu/kidney). Similarly, iBAK device combines two parts: hemofilter and bioreactor. The hemofilter is upgraded to incorporate silicon membranes with nanometer-scale slit-like pores to mimic the filtration function of the kidney’s glomeruli. These silicon membranes are fabricated using the microelectromechanical systems (MEMS) toolkit which enables the unprecedented control over nanoscale feature size and geometry in a scalable manufacturing process (Fissell et al., 2009). Importantly, to prevent coagulation of plasma in contact with silicon, the surface of the silicon membrane is modified to incorporate biomaterials which can greatly reduce the adhesion of proteins and platelets, complement activation, and coagulation (Fissell et al., 2009; Kanani et al., 2010; Melvin et al., 2010; Li et al., 2011; Muthusubramaniam et al., 2011). The hemofilter part helps filter the blood to remove waste products and toxins from blood. The other part, bioreactor, is infused with renal proximal tubule cells to rebalance electrolytes and other blood components. The bioreactor is upgraded from an earlier version of bioartificial renal epithelial cell system (BRECS) (Buffington et al., 2012). Like RAD, BRECS has human renal proximal tubule cells grown as confluent monolayer along the inner surface of a hemofiltration cartridge, but these renal proximal tubule cells are first expanded *in vitro* (Westover et al., 2012), and the whole system can be cryopreserved, enabling on-demand use for acute indications. Compared to BRECS, the upgraded bioreactor seeds expanded renal proximal tubule cells onto the silicon scaffold but not onto the surface of the traditional hemofiltration cartridge. These renal proximal tubule cells can reabsorb salts, sugar, and most water back to bloodstream and excrete toxins to the bladder. Instead of external electrical pump used in hemodialysis, this device uses the energy from patient’s own heart to pump blood through the hemofilter. Importantly, according to this group’s 2021 ASN conference abstract (# PO0513), this coffee cup-sized device has been implanted into pigs for preclinical evaluation. Within 3 days after implanting this device into the retroperitoneum of pig without any anticoagulation or immunosuppression treatment, the pig showed no severe side effect including hematoma, thromboembolism or other infection (peer-reviewed article has not been published at the time of writing). Importantly, one of the great advantages of this bioartificial kidney is that it doesn’t need blood thinning or immunosuppressant drugs, since the renal tubule cells in the device are separated from the recipient’s immune cells or antibodies as cells or larger molecules from the blood cannot pass through the nanopores of the hemofilter.

These results are encouraging, but some major challenges need to be solved for clinical development of this approach. First, to make the bioreactor component, large quantities of high-quality human proximal tubule cells are needed for manufacturing. In the current proof-of-concept studies, these cells are sourced from deceased donor human kidneys that are determined not suitable for transplantation. However, a reliable and sustainable source of human proximal tubule cells are urgently needed for future commercialization, which could potentially be provided from the directed differentiation of human pluripotent stem cells into renal epithelial cell types (see below). Second, long-term durability and efficacy of bioartificial kidneys upon transplantation remain to be determined: the nanopores in the silicon hemofilter may clog up over time, and it also remains unknown whether the proximal tubule cells maintain their differentiated phenotypes and functionality in the long run. Third, with the current design, the patients will rely on water and electrolyte supplementation and dietary restrictions to maintain the homeostasis. It would be a great improvement if a real-time biofeedback component can be incorporated to monitor the patient’s homeostatic state and regulate the function of the bioartificial kidney autonomously to maintain homeostasis.

## Recellularization—reseeding native kidney scaffold

Decellularized whole organ scaffold offers an attractive source for whole organ engineering. Decellularization can be achieved through perfusion at controlled pressure in an intact organ using detergents such as sodium dodecyl sulfate (SDS) or Triton X-100 to remove the cellular components of the organ (Guyette et al., 2014). Over the past decade, successful decellularization of the kidney has been reported, with successes from species including rat (Song et al., 2013; Caralt et al., 2015), pig (Orlando et al., 2012; Abolbashari et al., 2016; Poornejad et al., 2017), monkey (Nakayama et al., 2010), and human (Peloso et al., 2015). In general, after decellularization, few native cells remain in the kidney, leaving an acellular scaffold with retained vasculature and tissue structures, and extracellular matrix (ECM) components. ECM contains important mechanical, biophysical, and biochemical cues that regulate cell identity and function in the organ (Hussey et al., 2018). It is thus hypothesized that, if the acellular scaffold can be properly recellularized, it will be able to reconstitute a transplantable bioengineered organ.

Proof-of-concept recellularization of a decellularized kidney scaffold to make a transplantable bioengineered kidney was reported in 2013 from Ott and colleagues (Song et al., 2013). In this study, cadaveric rat kidneys were decellularized using renal artery perfusion with 1% SDS at a constant pressure of 40 mm Hg. Cells from two sources were then utilized to recellularize the kidney scaffold. Human umbilical venous endothelial cells (HUVECs) were infused through the renal artery and rat neonatal kidney cells (NKCs) were seeded through the ureter. After recellularization and additional 3–5 days of perfusion, HUVECs were found to line the vascular channels throughout the entire scaffold while NKCs were identified in the different compartments of the nephron, with podocytes appearing to preferentially engraft into the glomerular regions. Upon orthotopic transplantation, the bioengineered kidney perfused well without any evidence of bleeding from the vasculature. Importantly, urine is produced from the transplant although the volume is about 1/3 of that from a native kidney. Urinalysis, however, indicated only limited kidney function from the bioengineered kidney, reflected by much higher glucose and albumin but much lower creatinine and urea in the urine, compared with native kidney control. These results suggest that further optimization of recellularization is still needed to enhance the extent and distribution of the renal cell types across the kidney scaffold to improve the function of the bioengineered kidney.

Importantly, rapid progress in the areas of biomaterials and 3D bioprinting technologies (Murphy and Atala, 2014; Matai et al., 2020) have opened new avenues to fabricating scaffolds with designed geometrical, mechanical, biochemical, and biophysical features from either native or synthetic ECM biomaterials (Hussey et al., 2018). These new bioengineering tools might provide novel solutions to the current recellularization challenges. In addition to the optimization of recellularization, other challenges need to be overcome to move kidney organ decellularization/recellularization approach into a feasible therapy. The first consideration is the cell source for recellularization. Ideally a mixture of renal cell types representing different segments of the nephron, like the NKCs in the proof-of-concept study mentioned above (Song et al., 2013), should be used for recellularizing a kidney scaffold. For that, induced pluripotent stem cells (iPSC)-derived kidney organoids (see next section) might be able to provide such a source. Importantly, with iPSCs isolated from a transplant recipient’s own cells, in theory, it would be possible to recellularize an acellular kidney to avoid immune rejection. Second, most of the current proof-of-concept studies have been performed in rat models, but studies using human kidney scaffolds are still limited. More work will need to be done in decellularized human kidney scaffolds to show the viability of ECM scaffolding as well as proper transplantation and physiological function of a bioengineered kidney in large animal models.

## Stem cells—de novo generation of a kidney

Pluripotent stem cells (PSCs), including embryonic stem cells (ESCs) (Evans and Kaufman, 1981; Martin, 1981; Thomson et al., 1998) and induced pluripotent stem cells (iPSCs) (Takahashi and Yamanaka, 2006; Takahashi et al., 2007), have the potential to generate any cell type of the body (Robinton and Daley, 2012). With the recent progress in PSC-derived kidney progenitor cells and their derivative miniature kidney-like structures named “organoids,” PSCs hold great promise in rebuilding a kidney *de novo* by recapitulating normal kidney organogenesis.

Studies of kidney organogenesis have provided valuable insights into how we might be able to rebuild a kidney. Within UB and MM of the developing kidney, several self-renewing stem/progenitor populations drive the expansion and repetitive mutual induction of these structures. It is now clear that at least two progenitor populations are present in the MM, along with one in the UB (McMahon, 2016). *Wnt11* ^+^ UB progenitor cells (UPCs) reside at the branching tips of the UB epithelium (Majumdar et al., 2003; Rutledge et al., 2017); *Six2* ^+^ nephron progenitor cells (NPCs) are located in the inner layer of the MM, capping and contacting the UPCs (Self et al., 2006; Kobayashi et al., 2008); and *Foxd1* ^+^ interstitial progenitor cells (IPCs) are found in the outer layer of the MM, surrounding the NPCs (Kobayashi et al., 2014). In mice, lineage-tracing experiments have demonstrated that all of these progenitor populations can self-renew and differentiate, with NPCs giving rise to the nephrons, IPCs becoming the kidney stromal cell types and UPCs generating the CD network. In addition to these progenitor populations, it is thought that vascular progenitor cells (VPCs) form the blood vessels, for which less is known compared to other progenitors (McMahon, 2016). Importantly, of these progenitor populations, the interaction between NPCs and UPCs lays the foundation for kidney formation. During a typical UB branching cycle, UPCs self-renew upon receiving glial cell line-derived neurotrophic factor (GDNF) secreted from the surrounding NPCs (Durbec et al., 1996; Moore et al., 1996; Pichel et al., 1996; Sánchez et al., 1996; Treanor et al., 1996; Cacalano et al., 1998). Meanwhile, most of the NPCs self-renew and stay in close contact with the UPCs. Upon receiving Wnt9b signals from the trunk region of the UB, the NPCs farthest from the UPCs differentiate stepwise into pretubular aggregate (PA), renal vesicle (RV), S-shaped body (SSB), eventually forming a nephron tubule (Carroll et al., 2005). Importantly, the proximal nephron, which is the segment farthest from the UB, recruits endothelial cells to form vascularized glomeruli, which will filter the blood (Eremina et al., 2003; Eremina and Quaggin, 2004). The distal nephron closest to the UB fuses seamlessly with the UB epithelium to make a continuous lumen, so that the urine will pass from the nephron tubule to the UB-derived CD (Kao et al., 2012). This process repeats itself again and again, generating new nephrons, branching morphogenesis and more self-renewing NPCs and UPCs for the next cycle (Short et al., 2014). These progenitor cells are exhausted around the time of birth (postnatal day 2 for mouse; 36 weeks gestation for human), resulting in the limited regenerative potential of the adult kidney.

Considering the progenitors, as kidney’s building blocks, play central roles in kidney organogenesis, to recreate these embryonic/fetal-stage kidney progenitors from PSCs have become the focus of kidney researchers in the field of regenerative medicine in the past decade, which subsequently led to the creation of various kidney organoids that model kidney development and disease *in vitro*. Following normal developmental process, stepwise differentiation protocols have been established to generate NPCs from pluripotent stem cells, which can further generate 3D structures representing major segments of the nephron, including glomeruli, proximal tubule, loop of Henle and distal tubule (Taguchi et al., 2014; Morizane et al., 2015; Takasato et al., 2015). Similarly, UB-like cells have been generated from human PSCs in an earlier study (Xia et al., 2013), and recently, several groups have reported the generation of 3D branching UB organoids that have both UB tip and trunk segments (Taguchi and Nishinakamura, 2017; Mae et al., 2018), or have a near-pure population of the tip cells—the UPCs (Howden et al., 2020; Zeng et al., 2021). These UB organoids can further mature into CD organoids showing the expression of marker genes representative of principal cells and intercalated cells in the CD (Howden et al., 2020; Kuraoka et al., 2020; Tsujimoto et al., 2020; Zeng et al., 2021). Of note, taking a different approach than directed differentiation from PSCs, other groups have developed methods to culture and expand primary NPCs (Brown et al., 2015; Li et al., 2016; Tanigawa et al., 2016) and primary UPCs (Yuri et al., 2017; Zeng et al., 2021) that are isolated from embryonic/fetal kidneys, which can also differentiate into nephron and CD organoids, respectively. Importantly, Taguchi and Nishinakamura showed that when mouse embryonic stem cell (mESC)-derived NPCs and UB were mixed together, a self-organized higher-order kidney structure was generated, with the formation of both nephrons and CD in the same structure (Taguchi and Nishinakamura, 2017). Recently, this group further derived mouse IPC-like cells from mESCs and by mixing NPCs, UB, and IPCs, a more complete kidney structure was formed with the addition of stroma population to nephrons and CD (Tanigawa et al., 2022). Taken together, exciting progress has been made in the past decade, enabling us to produce large quantities of kidney progenitor cells and derive miniature kidney-like organoids from them, opening new avenues for kidney regeneration and disease modeling [comprehensively reviewed elsewhere (Little and Combes, 2019)]. However, despite the encouraging progress, it is still challenging to leap from progenitors or organoids to a transplantable organ. Several technical hurdles need to be overcome.

### Scalability

Patients can live well with at least 20%–30% of normal kidney function. Mice have an average of 14,000 nephrons per kidney, so they would need 5,600–8,400 nephrons to sustain life; in humans, with an average one million nephrons per kidney, a minimum of 400,000–600,000 nephrons are needed. However, in the miniature kidney organoid systems, dozens to hundreds of nephrons can be produced through self-organization. This number is far from adequate to sustain the life of mouse or human, so scale-up of the organoid system is required. For that, bioengineering approaches such as 3D bioprinting technologies (Lawlor et al., 2021) and a recently proposed biomanufacturing via organ building blocks (OBBs) approach (Wolf et al., 2022) will likely be useful.

### Plumbing

Most of the current kidney organoids recapitulate either the kidney’s nephron or CD, lacking a continuous nephron-CD structure to allow urine excretion. In the higher-order kidney organoid structure generated from mixing different types of mouse kidney progenitors (Taguchi and Nishinakamura, 2017; Tanigawa et al., 2022), the nephrons are interconnected with the CD system, allowing potential urine to drain. However, the system still lacks a single route for urine to exit. By incorporating bioengineering strategies to this system, this problem could potentially be solved in the future. It is also of note that, the higher-order kidney structures were generated using mouse PSCs, but a similar higher-order kidney structure has not yet been generated from human PSCs.

### Vasculature

For kidney to function, it is essential to have vasculature infiltrating properly into the glomeruli to generate primitive filtrate from the blood. In addition, peritubular vasculature is also important for performing reabsorption and excretion function of the kidney. However, the first generation of kidney organoids (Taguchi et al., 2014; Freedman et al., 2015; Morizane et al., 2015; Takasato et al., 2015) have very limited vasculature. Recent improvement via the manipulation of shear force and angiogenic signals (Homan et al., 2019), and the adjustment of embryonic patterning signals (Low et al., 2019), have greatly increased vasculature formation in the kidney organoids. However, the infiltration of vasculature into the glomeruli is still rare *in vitro*. In strong contrast, when kidney organoids were transplanted into recipient animals, blood vessels were formed and grafted well into the glomeruli of the transplant (Li et al., 2016; van den Berg et al., 2018; Garreta et al., 2019; Subramanian et al., 2019). Interestingly, these blood vessels were formed almost exclusively from the recipient’s own cells, paving the road for interconnecting the transplant to the circulation system of the recipient.

### Maturation

In-depth characterization of kidney organoids using single-cell sequencing technologies indicated the immature nature of the kidney organoids, resembling 1st–2nd trimester human fetal kidneys (Wu et al., 2018; Phipson et al., 2019; Subramanian et al., 2019), suggesting further maturation is needed for the kidney organoid to function properly. Interestingly, a recent study successfully generated highly patterned CD organoids that showed global gene expression profile similar to that of a postnatal kidney (Zeng et al., 2021), suggesting it is possible to further mature kidney organoid *in vitro*. Furthermore, transplanted organoids *in vivo* showed significantly improved maturity (van den Berg et al., 2018; Subramanian et al., 2019; Tran et al., 2019), suggesting the potential future use of host animals as incubators to achieve maturity and functionality of stem cell-derived kidney structures.

## Xenotransplantation—immunocompatible pig kidneys

Xenotransplantation opens the gate to an alternative source of transplantable kidney, from other animals. It also makes it possible to modify the donor, instead of treating the recipient with immunosuppressants, to overcome immune rejection. Naturally, the earliest trials were mostly using nonhuman primates (NHPs) as the kidney source, considering that they are evolutionary close to human. However, most of these trials were unsuccessful. In an early study from 1963, six patients received chimpanzee kidney xenotransplantation (REEMTSMA et al., 1964). Five of the xenotransplantation soon failed within 6 weeks from either rejection or infection. One patient did live a normal life for 9 months until eventually died possibly due to electrolyte disturbance.

Later, potential problems from using NHPs as the kidney source were noticed. These donor NHPs were mostly caught from wild that might carry infectious microorganisms or diseases. Furthermore, significant ethical issues remain, making NHPs less likely to serve as donors to solve the problem of organ shortage (Cooper et al., 2002). Pigs, on the other hand, seem to be a better option, considering human’s thousands of years of experiences in breeding them, their short period of time of maturation, and importantly, the high similarity in size, anatomy, and physiology between pig and human kidneys. In the 1990s, research focus was then turned to using pigs as xenograft donors. Nevertheless, transplantation of wild-type pig kidneys led to immediate hyperacute rejection, due to species differences.

To solve the problem, the focus has recently been shifted to genetically modify the donor pigs to generate pig strains with reduced immunogenicity. With new gene editing tools such as the CRISPR-Cas9 system (Jinek et al., 2012; Cong et al., 2013; Mali et al., 2013), it is now much easier to genetically manipulate pigs to accommodate our needs. Researchers have introduced human complement- and coagulation-regulatory proteins which significantly increased pig xenograft survival (Fodor et al., 1994; Cozzi and White, 1995; Wang et al., 2018a). On the other hand, knock-out of pig genes encoding pig xenoantigens also significantly increased pig xenograft survival as well (Kolber-Simonds et al., 2004; Yamada et al., 2005). Another problem for pig kidney xenotransplantation is the potential transferring of pathogenic microorganisms and porcine endogenous retrovirus (PERVs) to the human recipients. These problems can be solved by establishing pathogen-free breeding space (Cooper et al., 2020) and gene editing the donor animals to knock out PERV loci in the genome (Niu et al., 2017).

Pig-NHP xenotransplantation has been studied for decades which significantly contributed to our current understanding of xenotransplantation. However, success in NHPs does not predict the same outcome in humans. A model to further investigate pig-human xenotransplantation is needed. Recently, groups from New York University (Montgomery et al., 2022) and University of Alabama Birmingham (UAB) (Porrett et al., 2022) have both conducted pig-human xenotransplantation in brain-dead decedents using genetically modified pig kidneys. At NYU, a pig kidney with the knockout of a gene encoding α-Gal, a major antigen mediating hyperacute rejection response, was connected to the blood vessels from the upper leg and kept outside of the body. And at UAB, two pig kidneys harboring 10 genetic modifications, were transplanted into the decedent’s abdomen and connected to his bladder after his native kidneys were removed. In both cases, urine was produced normally and no hyperacute rejection was observed. Kidneys remained viable until termination of the studies after 54 and 74 h, respectively.

Although how physiology changes due to brain death might affect renal function remains unknown, these encouraging studies from NYU and UAB demonstrated that human decedent can be a good pre-clinical model to study safety and short-term effect of xenotransplantation, paving the road for establishing future clinical trials in living humans to evaluate the long-term safety and efficacy of this approach. In addition to hyperacute rejection, future long-term xenotransplantation studies will address the extent of acute rejections (Cornell et al., 2008) (including acute cellular rejection and acute humoral rejection) which would arise weeks to months after transplantation. Furthermore, common issues observed in current kidney allotransplant surgeries, such as renal allograft thrombosis (Ponticelli et al., 2009) and chronic rejections, will also need to be determined in the xenotransplants.

## Interspecies chimera—growing a human kidney in a pig

Interspecies chimera is an emerging approach combining stem cells, genome editing, and blastocyst complementation techniques to generate a targeted organ from the donor stem cells within the body of another species (Wu et al., 2016). Conceptually, it is hypothesized that, if PSCs from one species are injected into a mutant blastocyst of another species for which development of a specific organ is defective, leaving a niche for organ development, the injected PSCs-derived cells will compensate for the defect and form the missing organ. The success of this approach will make it possible to generate replacement human organs from animal hosts.

The proof of concept for the successful generation of PSC-derived organs *in vivo* via interspecies chimera was first reported by Nakauchi and colleagues in 2010 (Kobayashi et al., 2010), in which a rat pancreas was generated in a mouse. In these recipient mice, *Pdx1*, a critical gene encoding a transcription factor essential for pancreas development, was knocked out, exhibiting pancreas agenesis. Donor rat iPSCs labeled with EGFP were injected into *Pdx1* ^−/−^ mouse embryos at the blastocyst stage and the embryos were then transferred into the uteri of pseudo pregnant mice. Live mice were born, and their pancreas were entirely composed of EGFP-marked rat iPSC-derived cells. Interestingly, these pancreases were small, of the size of a mouse pancreas. In a follow-up study from the same group, a reverse experiment was performed in which mouse iPSCs were injected into *Pdx1* ^−/−^ rat blastocysts (Yamaguchi et al., 2017). This time the pancreases were entirely composed of the mouse iPSC-derived cells but were of the size of rat pancreases. Islets were prepared from the pancreas of the mouse-rat chimera and transplanted into streptozotocin-induced diabetes mouse models. Importantly, these transplanted islets successfully maintained normal glucose levels over 1 year in the absence of immunosuppression. These experiments provide proof-of-concept evidence for the potential therapeutic effects of iPSC-derived islets generated in interspecies chimera.

Similar strategies have been adopted to generate a kidney *in vivo* from PSCs using rodent intraspecies and interspecies chimera systems. Knockout of *Sall1*, a gene encoding a transcription factor that plays critical roles in the metanephric mesenchyme of a developing mouse kidney, led to kidney agenesis (Nishinakamura et al., 2001). However, when GFP-marked mouse PSCs were injected into *Sall1* ^−/−^ mouse blastocyst to form chimera, GFP + kidneys were formed (Usui et al., 2012). Further analysis confirmed that, the kidney’s nephrons and stroma were generated from the injected mouse PSC-derived cells, but not the kidney’s collecting duct, vasculature, or nerves that are not derived from metanephric mesenchyme and are thus not developmentally influenced by *Sall1* gene knockout. Importantly, these kidneys were grossly and histologically normal. In a follow-up study, loss-of-function *Sall1* mutant rats were generated using CRISPR-Cas9 genome editing tool and like *Sall1* ^−/−^ mice, anephric phenotype was observed in these *Sall1* mutant rats. Microinjection of mouse PSCs into *Sall1* mutant rat blastocysts successfully generated mouse-rat chimera with kidneys (Goto et al., 2019). Consistent with intraspecies chimera studies in *Sall1* ^−/−^ mice (Usui et al., 2012), various compartments of the nephron, including glomeruli, proximal tubule, loop of Henle and distal tubule, were entirely composed of GFP + mouse PSC-derived cells. Although the kidneys were histologically normal, both intraspecies and interspecies chimera did not survive to adulthood, likely due to the defect of milk suckling, another phenotype of *Sall1* gene knockout that affects the brain function, which might not be fully rescued in the chimera. This has prevented in-depth analysis of the functional maturation of the kidneys generated from the intraspecies and interspecies chimera.

Interspecies chimera provides a powerful platform to study xenogenic barriers and generate *in vivo* disease models. However, several major obstacles still prevent the translation of this approach into novel therapies to provide replacement human organs in the near future. One of the major issues is the difficulty of forming interspecies chimera using human PSCs. Human PSCs have been found to be able to incorporate into mouse (G afni et al., 2013; Takashima et al., 2014; Masaki et al., 2015), pig and cow (Wu et al., 2017) embryos at very early developmental stage—the inner cell mass (ICM) stage, but they failed to contribute to the development at later developmental stages, likely due to the species differences from dozens of millions of years of evolutionary distance between human and these species. To solve this problem, alternative human PSC states are actively being developed in anticipation that different pluripotent state may enhance chimera formation efficiency (Wu et al., 2015; Yang et al., 2017). Furthermore, forced expression of anti-apoptotic proteins in the PSCs has also been shown to increase interspecies chimera competency (Masaki et al., 2016; Wang et al., 2018b). These efforts along these lines might lead to novel breakthroughs in this area. In addition to technical obstacles to overcome to demonstrate feasibility of this approach, ethical issues and safety concerns from the public still remain, in terms of animal warfare and the likelihood of achieving human cognitive abilities, human-like appearance, or making human gametes, from the chimera (Wu et al., 2016).

## Conclusion and future perspectives

With the rapid technological development in the areas of regenerative medicine, genome editing, and bioengineering, over the past decade, several highly innovative approaches have been proposed aiming at providing solutions to the severe shortage of donor kidneys for transplantation. Here we have introduced the concepts of these approaches, summarized their current progress, and discussed potential challenges towards their future translation into novel therapies. It is of note that different approaches do not develop independently. Rather, these approaches can complement each other, and stem cell technologies appear to be at the center of these interconnections. For instance, the development of stem cell-derived kidney progenitors and organoids may be utilized to provide a reliable source of proximal tubule cells that are needed in commercializing the bioartificial kidney device. Large quantities of different kidney cell types that are required to recellularize a decellularized native kidney scaffold can also be provided from stem cell-derived kidney tissues. In addition, development of alternative PSC states with genetic modifications might overcome xenogenic barriers eventually leading to the generation of human organs in animal hosts through interspecies complementation. Taken together, in the past decade, we have seen remarkable progress in this area, and we are optimistic that novel kidney replacement therapies will likely benefit patients with kidney diseases in the next decade to come.
